# Animal Models for Henipavirus Research

**DOI:** 10.3390/v15101980

**Published:** 2023-09-22

**Authors:** Declan D. Pigeaud, Thomas W. Geisbert, Courtney Woolsey

**Affiliations:** 1 Galveston National Laboratory, University of Texas Medical Branch, Galveston, TX 77555, USA; ddpigeau@utmb.edu (D.D.P.); twgeisbe@utmb.edu (T.W.G.); 2 Department of Pathology, University of Texas Medical Branch, Galveston, TX 77555, USA; 3 Department of Microbiology and Immunology, University of Texas Medical Branch, Galveston, TX 77555, USA

**Keywords:** henipaviruses, animal models, Nipah virus, Hendra virus, zoonosis, vaccines, antivirals, monoclonal antibodies, pathogenesis, medical countermeasures

## Abstract

Hendra virus (HeV) and Nipah virus (NiV) are zoonotic paramyxoviruses in the genus *Henipavirus* (HNV) that emerged nearly thirty years ago. Outbreaks of HeV and NiV have led to severe respiratory disease and encephalitis in humans and animals characterized by a high mortality rate. Despite the grave threat HNVs pose to public health and global biosecurity, no approved medical countermeasures for human use currently exist against HeV or NiV. To develop candidate vaccines and therapeutics and advance the field’s understanding of HNV pathogenesis, animal models of HeV and NiV have been instrumental and remain indispensable. Various species, including rodents, ferrets, and nonhuman primates (NHPs), have been employed for HNV investigations. Among these, NHPs have demonstrated the closest resemblance to human HNV disease, although other animal models replicate some key disease features. Here, we provide a comprehensive review of the currently available animal models (mice, hamsters, guinea pigs, ferrets, cats, dogs, nonhuman primates, horses, and swine) to support HNV research. We also discuss the strengths and limitations of each model for conducting pathogenesis and transmission studies on HeV and NiV and for the evaluation of medical countermeasures.

## 1. Introduction

Hendra virus (HeV) and Nipah virus (NiV) are zoonotic pathogens found within the *Henipavirus* (HNV) genus (family *Paramyxoviridae*) that emerged in the 1990s [1,2,3]. Following the identification of HeV and NiV, other HNV members were later discovered, including Cedar virus [4], Mojiang virus [5], Ghana virus [6,7], and Langya virus [8]. Both HeV and NiV cause severe respiratory and neurological diseases in humans and certain livestock [1,2]. Fruit bats of the *Pteropus* genus are the natural reservoirs of NiV and HeV and replicating virus has been isolated in several bat species local to endemic regions of outbreaks [9,10,11]. The suspected natural hosts of Mojiang and Langya viruses are rats and shrews, respectively [5,8].

HeV first emerged in September of 1994, after several horses at a stable in Brisbane, Australia, developed a severe respiratory illness that was lethal in 65% of animals infected [12]. Two humans who came into direct contact with the fatally infected horses developed a severe influenza-like illness, which was lethal in one individual [1,12,13,14]. In 2004, 2008, and 2009, subsequent human cases occurred in veterinary or husbandry staff who came into contact with infected horses [15,16,17]. Additional equine outbreaks have occurred since then, but no other human cases have been reported. Since HeV’s emergence, there have been at least 63 spillover events in horses with an overall fatality rate of ~75% [18]. Only seven human cases of HeV have been recorded, 4 of which were fatal, and all occurred after direct contact with infected, symptomatic horses [19]. A second genotype of HeV (HeV-g2) was discovered in flying foxes [20] and horses [21]. HeV-g2 shares ~83% sequence homology to the prototype HeV. This genotype is lethal in horses, yet its pathogenicity in humans and nonhuman primates is presently unknown.

NiV was identified following an outbreak among pig farmers, first in Malaysia and then in Singapore during 1998 and 1999, resulting in 265 total cases and 105 deaths, corresponding to a mortality rate of 39% [2,3,22,23]. Epidemiological investigations early in these outbreaks found that many NiV-infected patients had direct contact with blood, tissues, or secretions of pigs suspected of NiV infection [2,3]. In Malaysia, as many as 93% of patients reported direct contact with swine in the two weeks prior to developing symptoms of NiV encephalitis [2]. A case-control study identified that close contact with pigs was the primary source of the NiV outbreak in Malaysia [24]. Furthermore, this initial outbreak of NiV in Malaysia and Singapore was quelled by the culling of an estimated 1 million pigs in Malaysia and the cessation of pig exports from Malaysia to Singapore [22]. Overall, the total number of NiV cases recorded exceeds 650, and the continual emergence of the virus in Bangladesh and India increases these numbers almost annually [25].

The ominous potential of NiV was swiftly realized as it manifested as febrile encephalitis and severe respiratory disease in humans, with case fatality rates (CFR) of up to 100% in certain outbreaks [26,27,28]. Genetic analysis has identified at least two strains of NiV responsible for outbreaks in different geographical areas: NiV-Malaysia (NiV-M) and NiV-Bangladesh (NiV-B) [29,30]. NiV-M and NiV-B share ~92% nucleotide sequence homology [30] and may exhibit divergent pathologies in humans [23,31]. The Malaysia strain caused the initial outbreak of NiV from 1998–1999 in Malaysia and Singapore and resulted in a high percentage of patients with neurological disease, with most encephalitic cases correlating with lethality in these outbreaks [26,27,28]. An additional outbreak of the Malaysia strain in the Philippines in 2014 was associated with a ~82% CFR in patients presenting with acute encephalitis syndrome [32]. The Bangladesh strain of NiV has caused repeated and near-annual outbreaks in Bangladesh and India between 2001 and 2023 [27,28,33,34]. The outbreaks caused by NiV-B have had higher CFRs, averaging about 75% [31], with human-to-human transmission also observed [35,36]. A 2018 outbreak of NiV-B in India resulted in 21 deaths among 23 total cases (91% CFR) [33], and a current outbreak of NiV-B in Bangladesh has resulted in 11 cases with eight deaths [34]. Subsequent outbreaks of NiV-B that have occurred in Bangladesh and/or India have reported a higher proportion of respiratory disease observed than in Malaysia [31,37]. Although imprecise characterizations have distinguished the Malaysia strain for causing neurological disease and the Bangladesh strain for causing pulmonary disease, it is important to note that both strains inflict pulmonary and neurological disease in both humans and animal models.

Viral entry of HNVs is initiated following binding of the attachment (G) glycoprotein to host-cell Ephrin-B2 or Ephrin-B3 ligands, after which the fusion (F) glycoprotein mediates membrane fusion between the viral and host-cell membranes, enabling viral entry to the cell [38,39]. Ephrin B2 is present in various cell types, including neurons, endothelial cells, smooth muscle surrounding arteries, placental tissue, spleen, and the lining of lymph nodes’ sinuses. In contrast, ephrin B3 is predominantly expressed in the central nervous system (CNS), particularly the spinal cord midline, and functions to prevent corticospinal tract axons from recrossing the midline [40]. Ephrin B3 is also expressed on lymphoid cells, which could potentially explain the occurrence of acute lymphoid necrosis induced by NiV infection; while lymphocytes are not directly permissive to NiV, the virus can bind these cells as a vehicle for spread into tissues [38,41]. The extensive species tropism of HNVs is primarily attributed to the conserved protein sequence of ephrin B2 and B3 across numerous species [42]. Despite this high conservation, HNVs do not cause disease in immunocompetent mice after intraperitoneal challenge [43]. Hamsters and ferrets have been the most valuable small in vivo models, particularly for triaging candidate vaccines and treatments, but these fail to reflect some major aspects of NiV disease in humans [44]. The animal model that appears to reproduce human NiV infection most faithfully is African green monkeys (AGMs) [44,45], which we will discuss in detail below.

**Figure 1:**
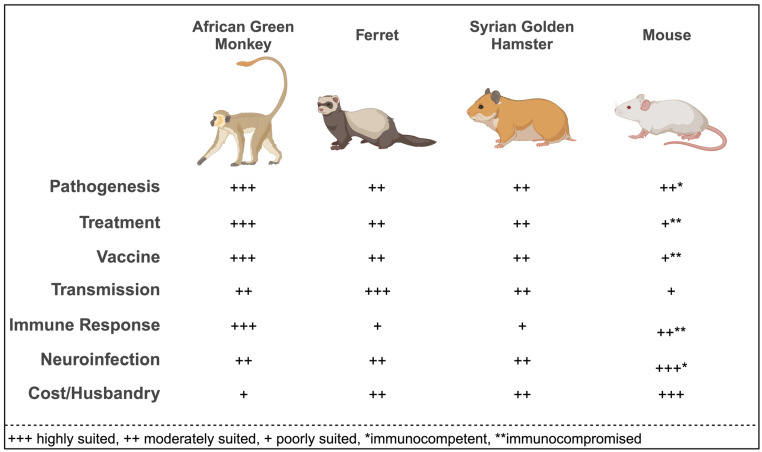
General suitability of AGMs, Ferrets, SGH, and Mouse models by study type.

This review comprehensively evaluates the suitability of current animal models for conducting studies on the pathogenesis and transmission of HeV and NiV and assessment of experimental countermeasures. A detailed examination of the strengths and limitations of each model is offered to facilitate an informed selection of the optimal model for the intended research purpose. Figure 1 describes the general suitability of several animal models for specific research objectives.

## 2. Henipavirus Infection in Humans

### 2.1. Hendra Virus

HeV infection in humans produces an acute respiratory and encephalitic disease. At present, seven human patients are confirmed to have been infected with HeV, five of whom died. The incubation period ranges from as few as four days to two weeks following exposure. All cases initially exhibit non-specific, flu-like symptoms of fever, cough, myalgia, nausea, and lethargy, which subsequently progress to acute respiratory distress and encephalitis in fatal cases [13,15,16]. Findings in human cases of HeV infection included pneumonitis, respiratory failure, renal failure, and arterial thrombosis [15]. Neurological signs and symptoms are presented as headache and confusion, and, in fatal cases, tonic-clonic seizure and ataxia [13]. Notably, a single case of fatal relapsing encephalitis occurred approximately 13 months after acute signs of HeV disease resolved [46]. Importantly, no human-to-human transmission of HeV has been observed.

### 2.2. Nipah Virus

Clinically, the incubation period for NiV infection ranges from a few days to two weeks [23,31]. NiV can cause atypical pneumonia [23] or necrotizing alveolitis with hemorrhage, pulmonary edema, and aspiration pneumonia [47], leading to acute respiratory distress syndrome. The pulmonary syndrome is presented as an influenza-like illness, with hypoxemia and diffuse alveolar shadowing in chest X-rays. NiV also causes severe encephalitis with cognitive, sensory, and motor neurological signs and symptoms [22,23,31,48]. Clinical signs reported in NiV encephalitic syndrome include areflexia, hypotonia, abnormal pupillary and doll’s eye reflex, tachycardia, hypertension, myoclonus, meningism, convulsions, and seizures. Brain MRI scans in acute NiV encephalitis show disseminated, small, discrete hyperintense lesions in both grey and white matter. Targeting endothelial cells by NiV leads to systemic vasculitis and infection of multiple organ systems, including the kidneys, liver, heart, and brain [49,50]. Pulmonary or encephalitic disease can be lethal, and many survivors of encephalitic infection face long-term neurological sequelae [51]. Late-onset and relapsing encephalitis due to prior NiV infection has been reported months to years after initial infection, with the longest time to relapse described so far being 11 years [52,53,54].

## 3. Animal Models of HNV Infection—Initial Studies

The first animal challenge studies with HeV (previously equine morbillivirus) were performed in healthy adult horses to reproduce the severe respiratory disease observed during the initial outbreak [1,12,13]. The experiments successfully recapitulated clinical signs of disease seen in natural infection, including anorexia, fever, and respiratory distress, with the primary accompanying pathology being pulmonary edema and histological evidence of syncytial cell formation of vascular endothelium across lung, brain, spleen, heart, and kidneys [1]. Shortly after the initial equine experiments, a pilot study was performed to identify the susceptibility of several common laboratory species, including mice, guinea pigs, rats, chickens, rabbits, cats, and dogs with 5 × 10^4^ TCID_50_ of HeV administered intraperitoneally (i.p.) [55]. Only in guinea pigs and cats were clinical signs of disease observed. A wide range of species were then investigated for suitability as models of HNV disease.

## 4. Mice

The mouse model for HNV disease has not been widely used in the field for studies of pathogenesis or medical countermeasures due to initial challenge experiments failing to produce clinical disease in immunocompetent mice (Table 1). Recent studies employing transgenic mice with modifications aimed to dampen the host antiviral immune response have yielded positive findings, suggesting that the model may be of utility if further refined (Table 2). A mouse model for HNV disease would be highly useful due to plentiful reagent availability, ease of handling, space requirements in Biosafety Level (BSL)-4 containment, and low cost.

### 4.1. Immunocompetent Mice

Initial challenge studies employing immunocompetent, juvenile mice (Table 1) did not result in clinical disease or seroconversion when inoculated i.p. with 5 × 10^3^ TCID_50_ of HeV-prototype or 1 × 10^7^ PFU of NiV-M [43,55]. Mice surviving infection with NiV were re-challenged and developed an antibody response but still did not show clinical signs of disease [43]. A later study demonstrated that intracerebral (i.c.) NiV-M infections with 1 × 10^5^ PFU were 100% lethal by day 5 post-infection in C57BL/6 mice, whereas i.p. inoculation with a 1 × 10^6^ PFU dose in this model did not result in disease [58]. A subsequent study examining the susceptibility of juvenile (8-week-old) and aged (12-month-old) BALB/c and C57BL/6 mice to HeV found that a 5 × 10^4^ TCID_50_ dose administered intranasally (i.n.) was lethal in aged mice of both genetic backgrounds [56]. This study also examined subcutaneous (s.c.) HeV challenge (5 × 10^4^ TCID_50_), but mice did not exhibit clinical signs, and only a small minority developed a neutralizing antibody response. In contrast, juvenile BALB/c and C57BL/6 mice did not develop clinical signs of disease; however, histological assessment of asymptomatic mice did uncover encephalitic lesions and viral antigen deposition. Clinical signs of disease observed in aged mice challenged with HeV in this study were largely neurological, with depression, ataxia, hypersensitivity, and tremors reported, with mice succumbing to disease or meeting euthanasia criteria between 11- and 21-days post-infection. Encephalitis characterized by neuronal degradation, perivascular cuffing, and non-suppurative meningitis was identified in all C57BL/6 mice showing clinical signs of disease [56]. Antigen deposition was detected in the lungs of animals, and live virus was re-isolated in BALB/c mice; however, clinical signs of respiratory distress were not observed, and no histological lesions were present in major target organs of HeV infection [56].

### 4.2. Immunodeficient Mice

The susceptibility of immunocompromised mice has also been evaluated, with type I interferon receptor knockout (IFNAR-KO) mice demonstrating uniform lethality for both NiV and HeV (Table 2) [58]. I.p. infection with 1 × 10^6^ PFU in 3– (HeV) and 9–10-week-old (NiV) IFNAR-KO mice achieved uniform lethality [58]. Groups of 3- and 12-week-old IFNAR-KO mice were next i.n. challenged with 1 × 10^6^ PFU of HeV, but neither group developed clinical disease. Nevertheless, HeV genomic RNA was detected in the brain at levels lower than groups challenged i.p. [58]. Interestingly, immunocompetent mice supported higher levels of HeV RNA in the brain following i.n. infection as compared to those inoculated via the s.c. route at an identical challenge dose [56]. Among many potential reasons, this disparity may be attributed to the i.n. route delivering more infectious particles in close anatomical proximity to the olfactory bulb. Not only was i.n. challenge lethal in aged immunocompetent mice, but the challenge dose was also lower than that used in the IFNAR-KO mice [56,58]. Taken together, these findings indicate that lethality is both dose- and route-dependent. It is unclear how lethality was higher in immunocompetent mice inoculated with a lower dose than that used in the IFNAR-KO mouse model. These discrepancies warrant further investigation.

IFNAR-KO mice infected i.p. with NiV-M showed clinical signs consistent with neurological disease, including head tilt and locomotor disabilities that progressed to paralysis in the late stages of infection. Breathing difficulty was seen only in some i.n. challenged animal. Other clinical signs included hunched posture, decreased grooming, and weight loss. The LD_50_ for NiV-M in IFNAR-KO mice inoculated via the i.p. route was determined to be 8 × 10^3^ PFU. Transgenic mice deficient in multiple innate pattern recognition signaling adaptor proteins including myeloid differentiation primary response 88 (MyD88), TIR-domain-containing adapter-inducing interferon-b (TRIF), mitochondrial antiviral-signaling protein (MAVS), and stimulator of interferon genes (STING) (MyD88/TRIF/MAVS/STING-KO) were also assessed for susceptibility to NiV infection. These adaptor molecules are implicated in the antiviral sensing activities of toll-like receptors (TLRs) and RIG-I-like receptors (RLRs), which detect DNA and RNA typically associated with viruses and their replication cycle. MyD88/TRIF/MAVS/STING-KO mice developed lethal disease following i.p. challenge with 1 × 10^6^ PFU of NiV-M, and clinical signs were consistent with those observed in IFNAR-KO studies [61]. Principal clinical signs aligned with a neurological-skewed disease course as hunched posture, prostration, and paralysis were observed, with all (MyD88/TRIF/MAVS/STING-KO) subjects succumbing to disease by 11 days post-infection [62]. Gross or histopathologic lesions were not described by the authors of this study; however, antigen was detected in the brain.

**Table 2:** Experimental findings in immunodeficient mouse models of HNV Disease.

Species/Genetic Background (Age)	Virus (Isolate)	Dose	Route	% Lethality	Clinical Disease	Reference
IFNAR-KO (3-week-old)	HeV (prototype)	1 × 10^6^ PFU	i.p.	100%	Weight loss, aggressiveness, prostration, paralysis	[58]
IFNAR-KO (6-week-old)				~80%		
IFNAR-KO (18-week-old)				50%		
IFNAR-KO		1 × 10^6^ PFU	i.n.	None	None	
NOD/SCID/γcnull	NiV-M	1 × 10^5^ TCID_50_	intra-graft	None	None	[63]
		1 × 10^5^ TCID_50_	i.d.			
IFNAR-KO	NiV-M (UMMC1)	1 × 10^6^ PFU	i.p.	100% (5/5)	Weight loss, aggressiveness, prostration, paralysis	[58]
		1 × 10^6^ PFU	i.n	60% (3/5)		
		1 × 10^5^ PFU	i.c.	100% (5/5)		
	^a^ rNiV-Fluc^NP^	8 × 10^4^ PFU	i.n.	50% (2/4)	Hunched posture	[64]
		8 × 10^5^ PFU	i.p.	100% (4/4)	Hunched posture, lethargy, ataxia, seizure	
C57BL/6 MyD88/MAVS-KO	NiV-M (UMMC1)	1 × 10^6^ PFU	i.p.	66% (4/6)	Lordosis, prostration, paralysis	[61]
C57BL/6 IFNAR-KO		1 × 10^6^ PFU		83% (5/6)		
C57BL/6 MyD88/TRIF/MAVS/STING-KO	NiV-M (UMMC1)	1 × 10^6^ PFU	i.p.	100% (5/5)	Yes, not described	[62]
C57BL/6 MyD88/TRIF/MAVS-KO		1 × 10^6^ PFU		40% (2/5)		
C57BL/6 IFNAR-KO		1 × 10^6^ PFU		80% (4/5)		
C57BL/6 MyD88 KO		1 × 10^6^ PFU		None	None	
C57BL/6 MyD88/TRIF-KO		1 × 10^6^ PFU		None		
IFNAR-KO (37–44-day-old)	NiV-M	1 × 10^4^ TCID_50_	i.p.	50% (4/8)	Clinical signs including weight loss ^b^	[59]
		1 × 10^6^ TCID_50_		63% (5/8)		
		1 × 10^7^ TCID_50_		75% (6/8)		

^a^ Recombinant NiV containing a luciferase reporter gene. ^b^ Comprehensive numerical clinical scores were described in this study, with individual signs not specified for subjects.

Currently, no published studies employ mice as a model to study antiviral countermeasures to HNV infection. The use of immunocompromised mouse strains clouds interpretations of the immune response to HNV infection or medical countermeasures. Additional challenge studies should be performed to refine the challenge route and dose across multiple genotypes. Since intranasal inoculation of HeV is uniformly lethal in immunocompetent mice, determination of LD_50_ should be conducted. Because of the relevancy to NiV pathogenesis and transmission, aerosol challenge studies should also be carried out. Lastly, to detect pathogenicity differences, challenge studies should be performed with multiple strains, including NiV-B, the strain responsible for the most frequent, recent, and lethal outbreaks in India and Bangladesh [33,34].

## 5. Syrian Golden Hamster

The Syrian golden hamster (SGH; *Mesocricetus auratus*) model is the most common rodent model employed in HNV pathogenesis, prophylaxis, and treatment studies. Both HeV and NiV infections produce disease in SGHs that is highly similar to that observed in human cases. The SGH model was first developed for NiV in 2003 [43] and HeV in 2009 [65]. Following these initial studies, continued investigation of challenge route, dosing, and HNV strains has enabled refinement of the model (Table 3). The SGH model has been used to evaluate the in vivo efficacy of antivirals, including favipiravir [66], ribavirin [67], and chloroquine [68] against lethal NiV challenge. Experimental vaccine countermeasures to HNVs spanning a variety of platforms, including ChAdOx1 [69], virus-like particle [70], mRNA [71], and recombinant vesicular stomatitis virus [72] based vaccines, have been described. Monoclonal antibody [73] and passive immune sera treatment studies [74] have also been studied in this model.

SGHs are highly susceptible to lethal HeV infection, with an LD_50_ calculated to be 12 PFU in seven-week-old animals challenged via the i.p. route [65]. Inoculation with doses as low as 1 × 10^3^ PFU i.p. resulted in death within six days of challenge [65]. Unlike humans and AGMs, the Bangladesh strain is less pathogenic than the Malaysia strain in hamsters [79]. HeV infection of SGHs resulted in acute respiratory disease marked by labored breathing and diffuse pulmonary infiltrates on thoracic radiography alongside neurological signs, including paralysis, limb trembling, and seizure [65,78]. Antigen was detected in the brain, lungs, heart, kidney, liver, and spleen as well as within blood vessel walls within 2–3 days of i.p. infection with 1 × 10^3^ PFU of HeV [65]. A higher challenge dose (1 × 10^5^ PFU) of HeV produced more severe lesions in the same organs. The challenge dose and route of HeV in the SGH model results in varying clinical signs and disease courses, with high-dose i.n. challenge producing a more severe respiratory disease state and rapid death, and low-dose producing a more neurological skewed disease course [78]. The lethality of HeV infection in SGH does decrease in response to the age of the hamster, with a dose of 100 LD_50_ being completely lethal in seven-week-old hamsters but only ~84% lethal in 11-week-old hamsters [65]. Furthermore, while seven-week-old hamsters all died by day 5 post-infection, fatally infected SGHs in the 11-week-old group died by day 12 post-infection. The potentially decreased sensitivity to lethal HeV infection with increased age must be accounted for in vaccine studies, as prime-boost dose regiments may require higher challenge doses to achieve uniform lethality in control animals.

NiV infection of SGHs results in respiratory and neurological signs of disease consistent with human infection, including acute interstitial pneumonia, systemic vasculitis, encephalitis, and death. Uniform lethality is achieved through multiple challenge routes, including i.n., i.p., and small particle aerosol. A number of experiments have been performed to elucidate the LD_50_ of NiV-M and NiV-B delivered i.p. and i.n., and there is some variation in these values [79,83]. As was seen with the HeV challenge, the disease severity of NiV-infected SGH is route and dose-dependent, and there are differences in pathogenicity observed between NiV-M and NiV-B. Initial studies determined the LD_50_ of NiV-M in SGH to be 2.7 × 10^2^ PFU and 4.7 × 10^4^ PFU via the i.p. and i.n. routes, respectively [43]. Doses of 1 × 10^4^ PFU or 1 × 10^3^ TCID_50_ are uniformly lethal when delivered via the i.p. route. Lethality is more reliably achieved with lower doses via the i.p. route. Time-to-death also varied depending on the challenge route, with i.p. challenged animals succumbing to the disease within 5–9 days post-infection and 9–15 days for i.n. [43]. A recent study sought to robustly determine whether differences in lethality, disease course, and time to death varied between i.n. and i.p. challenge routes. This report found that the i.p. challenge disseminated the virus more rapidly to tissues and that there was no difference in survival when the same dose was delivered [83]. Initial dose susceptibility employing serial dilutions of NiV-M ranging from 1 × 10^4^ to 1 × 10^6^ TCID_50_ i.n. and 1 × 10^3^ to 1 × 10^5^ TCID_50_ i.p. were uniformly lethal; however, a dose of 1 × 10^2^ TCID_50_ i.p. resulted in 1 of 4 animals surviving, which is consistent with other studies [79,83]. The challenge of SGHs with 1 × 10^2^ TCID_50_ of NiV-B delivered i.p. resulted in 100% survival in a separate study by a different group [79]. Challenge of SGH via the i.n. route produces a respiratory-skewed disease, and i.p. challenge results in more neurological signs. Lesion severity in the lungs diverges at the early stages of the disease. Severe broncho-interstitial pneumonia and thickening of alveolar walls occurred at days 2 and 4 post-infection, whereas i.p. challenged animals showed only mild interstitial pneumonia [83]. Viral RNA was detected in high abundance starting at day 2 post-infection in i.n. challenged subjects; however, no RNA was observed at this time point in i.p. challenged animals. This is likely because the i.n. delivery ensures large amounts of virus travel deep into the lungs, producing acute and severe respiratory disease. Meningitis was observed only in i.p. challenged animals, and no viral RNA was found in the brain of i.n. challenged animals [83].

The challenge of SGHs with whole-body aerosols containing NiV-M at doses of 1 × 10^2^ to 1 × 10^5^ PFU resulted in uniform lethality at the highest dose, with an estimated LD_50_ of less than 1 × 10^2^ PFU [80]. Animals exhibited clinical signs of disease starting five days post-infection, including weight loss and decreased grooming, which progressed to severe respiratory and neurological signs of disease. The mean time-to-death occurred around seven days post-infection for animals receiving a 1 × 10^5^ PFU dose [80]. Prominent pathological findings included vasculitis of medium and large blood vessels, edema, broncho-interstitial pneumonia, and mild meningitis. As seen with other routes of challenge, lower challenge doses resulted in incomplete lethality and extended disease courses. Notably, there was no correlation between dose and whether the infection produced a neurological or respiratory-focused disease, as animals in all dose groups demonstrated a mix of respiratory and neurological disease. These studies suggest that the aerosol challenge of NiV in the SGH model is useful for evaluating medical countermeasures against potential natural routes of exposure.

There is no evidence of HeV transmission between SGHs through direct contact or aerosols. Naïve animals co-housed with HeV-infected subjects did not show clinical signs of disease or seroconvert by study endpoint [65]. Similar studies evaluated viral shedding and transmission of NiV via direct contact, aerosol, and fomites. Virus shedding detected by titration was identified in oral swabs with doses as low as 1 × 10^3^ TCID_50_ i.n.; however, the virus was detected in oral, nasal, rectal, and urogenital swabs from animals inoculated with a higher dose of 1 × 10^7^ TCID_50_ of NiV-M [77]. Naïve SGHs exposed to aerosols from NiV-M infected subjects did not seroconvert nor exhibit clinical signs of disease. However, viral shedding was detected by qRT-PCR in naïve SGHs co-housed with NiV-M infected subjects or those exposed to bedding, food, and water from infected animals [77]. None of the naïve animals in the fomite or aerosol transmission group seroconverted by study endpoint; however, several animals from the direct contract transmission group did, which is consistent with other direct-contact NiV transmission experiments in the SGH model [43]. Foodborne transmission of NiV has also been investigated; ~63% of SGHs drinking simulated date palm sap contaminated with 5 × 10^8^ TCID_50_ of NiV-B developed neurological disease [85].

## 6. Guinea Pig

The guinea pig (*Cavia porcellus*) was among the first rodents to be experimentally infected with Hendra virus (Table 4) [55]. Four out of five guinea pigs challenged via the s.c. route with 5 × 10^4^ TCID_50_ of HeV succumbed to disease 8–13 days post-infection after clinical signs of acute respiratory distress were observed [55,86]. Only one of twelve guinea pigs challenged i.n. with 3 × 10^4^ TCID_50_ of HeV showed clinical signs of dyspnea and was euthanized; however, all asymptomatic animals seroconverted and remained clinically well through the study endpoint [87]. Intradermal inoculation also failed to produce clinical disease, and animals did not seroconvert [87]. The guinea pig model recapitulated the systemic vasculitis seen in human HeV infection, with principal histological findings including syncytial cell formation in vascular endothelium, fibrinoid degeneration of arteries and veins, and thrombi [86,87]. Vasculitis in the lungs, heart, kidney, urinary bladder, and brain was marked by infiltration of mononuclear cells, eosinophils and fibroblasts into vessel walls and surrounding structures. In contrast to the disease seen in humans and horses, signs of severe pulmonary edema or acute encephalitis were absent. Pregnant guinea pigs challenged i.p. with 5 × 10^4^ TCID_50_ of HeV showed clinical signs consistent with previous studies, yet infection was abortive in one subject, and HeV was re-isolated from fetal tissues [88]. Placental tissue also showed multi-focal necrosis and vascular degeneration.

Limited studies have been performed to establish guinea pigs as a model for NiV disease. An initial study showed guinea pigs i.n. infected with 6 × 10^5^ PFU of NiV-M did not result in clinical disease or seroconversion by the study endpoint, and challenge with 1 × 10^7^ PFU i.p. resulted in only low fever and weight loss [43]. The lack of clinical disease in guinea pigs challenged i.n. with NiV or HeV indicates that this challenge route is not conducive to productive infection. Guinea pigs challenged with a lower i.p. dose of 5 × 10^4^ TCID_50_ produced similarly mild clinical signs, including ruffled fur and slight ataxia 7–9 days post-infection [90]. A subsequent study in guinea pigs exposed to 6 × 10^4^ PFU of NiV-M resulted in severe clinical signs of disease and 92% lethality within 4–8 days post-infection [91]. Histopathological lesions were present in guinea pig lung, spleen, brain, bladder, uterus, kidney and lymph nodes, and consisted of epithelial syncytial cell formation and fibrinoid necrosis. It is unclear why 1 × 10^7^ PFU i.p. did not result in clinical signs, but 6 × 10^4^ PFU caused nearly uniformly lethal disease. Except for the ataxia reported in one guinea pig challenged with 5 × 10^4^ TCID_50_, clinical neurological signs are not reported for NiV challenge in guinea pigs. However, histopathological lesions can include viral and eosinophilic inclusions, meningeal vasculitis, and lymphocytic meningitis [91]. The most severe lesions are found in the urogenital tract. Further studies are needed in this model to determine appropriate infective doses and whether NiV-B produces similar disease.

## 7. Ferret

The domesticated ferret (*Mustela putorius furo*) is a well-characterized model that effectively recapitulates human NiV and HeV disease (Table 5). In ferrets, infection with HNVs through the combined intranasal/intratracheal (i.t./i.n.) or oronasal (o.n.) route leads to the onset of fever between days 4 and 7 post-challenge [92,93]. This fever progresses rapidly, resulting in severe respiratory distress and neurological signs appearing between days 6 and 10, depending on the challenge dose. Clinical manifestations in ferrets include coughing, nasal discharge, dyspnea, cerebral edema, tremors, and partial or complete limb paralysis. Unlike hamsters, there is no observable connection between the challenge dose and the clinical outcome in ferrets.

**Figure 2:**
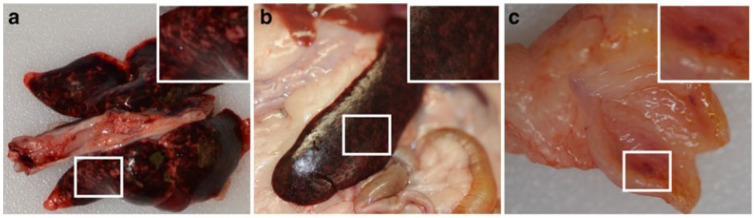
Representative gross pathology of lung, spleen, and urinary bladder from ferrets challenged with rNiV-M. Adapted from [101]. Representative gross pathology of the lung (**a**), spleen (**b**) and urinary bladders (**c**) taken from ferrets infected with NiV-M (**a**–**c**). Inserts show magnified regions of each specimen. Findings included multifocal to coalescing hemorrhage and necrosis of all lung lobes (**a**), splenomegaly and multifocal necrosis in the spleen (**b**), and large hemorrhagic lesions in the urinary bladder (**c**).

Gross pathological changes mainly manifest within the respiratory tract and include scattered pinpoint hemorrhagic lesions throughout the lung surface (Figure 2). These lesions stand out as they are notably different from the extensive hemorrhagic lesions found in hamsters and AGMs [78,103,104]. Histopathological alterations are primarily concentrated in the lungs, spleen, and kidneys [92] and include acute focal necrotizing alveolitis and pulmonary vasculitis, as well as necrosis in the spleen and kidneys. While vasculitis and encephalitis are typically absent, occasional instances of nonsuppurative meningitis can be observed. The presence of viral antigen is detected in syncytial cells of small blood vessels and alveolar walls in the lungs, along with necrotic glomerular and tubular epithelium in the kidneys. Despite minimal histopathological changes in the brain, HNV antigen is identified in meningeal blood vessels, choroid plexus endothelium, and neurons. Antigen detection is noted in various tissues, including the respiratory tract, brain, liver, spleen, and kidneys [92,98].

The ferret model has been utilized to compare potential distinctions in transmission and pathogenesis between the Malaysia and Bangladesh strains of NiV [98]. Throughout the infection course in ferrets, notably higher virus levels are retrieved from oral secretions in animals infected with NiV-B compared to NiV-M. Nevertheless, no endeavors have been undertaken to study inter-animal transmission in this model. Beyond transmission and pathogenesis investigations, ferret models have also proven effective in evaluating vaccines and therapeutics [92,93,94]. The main disadvantage of the ferret model is that limited reagents exist to conduct advanced immunological analyses.

## 8. Nonhuman Primates

Experimental challenge studies of NiV and HeV have been performed in a diverse array of nonhuman primates species, AGMs (*Chlorocebus aethiops*) [103,104], cynomolgus macaques (*Macaca fascicularis*) [105], and the common marmoset (*Callithrix jacchus*) [106]. Lethal infection is reliably achieved in AGMs across multiple routes that simulate natural routes of exposure, including inhaled small particle aerosol and i.t./i.n. inoculation. Conversely, infection of cynomolgus macaques results in mild to no disease or inconsistent lethality [105]. AGMs are ideal for evaluating promising medical countermeasures to NiV and HeV as they faithfully recapitulate the respiratory and neurological disease observed in human cases.

### 8.1. African Green Monkey

As mentioned, the AGM model is the gold standard for both NiV and HeV infection, as it accurately recapitulates human pathogenesis and is amenable to multiple challenge routes, including aerosol. Moreover, a wide range of immunological reagents are available for detailed characterization of the host response [105,107].

The challenge of AGMs i.t. with 4 × 10^5^ TCID_50_ of HeV resulted in a uniformly lethal disease course highly similar to that observed in humans with animals succumbing to disease or meeting euthanasia criteria 7–9 days following the challenge (Table 6) [104]. Clinical signs of disease became apparent 5–6 days after infection and included frothy nasal discharge, labored breathing, mild depression, seizures, muscle fasciculations, and radiology depicting pulmonary congestion and consolidation by days 7 and 8, indicative of interstitial pneumonia [104].

**Figure 3:**
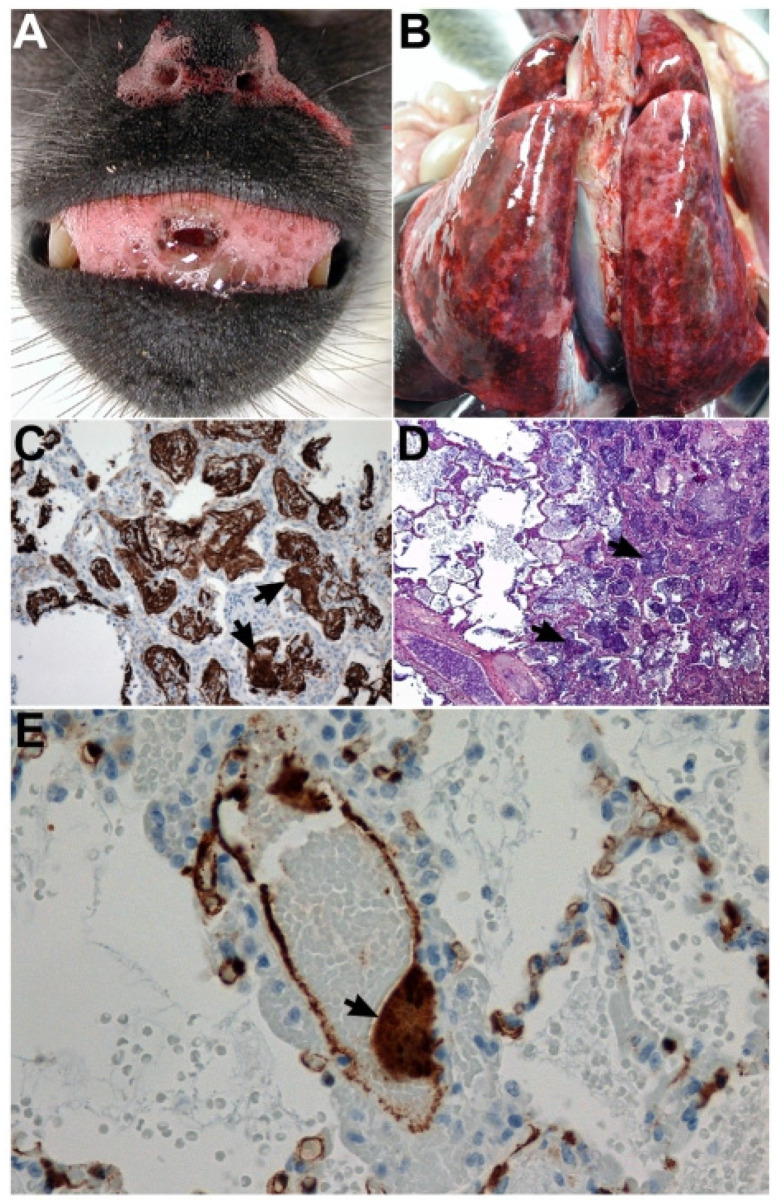
Representative pathogenesis of experimental NiV infection in the African Green Monkey, adapted from [103]. Panels (**A**,**B**,**D**,**E**); AGM euthanized nine days post-challenge with 1.3 × 10^6^ PFU of NiV i.t./o.n. Panel (**C**); AGM succumbed to infection 11 days post challenge with 8.1 × 10^4^ PFU of NiV i.t./o.n. (**A**) Serosanguineous, frothy oronasal exudate. (**B**) Wet, heavy, congested, and hemorrhagic lungs. (**C**) Lung, right diaphragmatic lobe by immunohistochemical (IHC) stain depicting fibrin deposition in and around alveolar spaces (arrows); 40× magnification. (**D**) Lung, right diaphragmatic lobe by PTAH connective tissue stain showing an abundance of polymerized fibrin in and around alveolar spaces (arrows); 100× magnification. (**E**) Localization of NiV antigen by IHC stain within a lung blood vessel with endothelial syncytia (arrow) and immunopositive cells (brown) abundant in alveolar septae; 400× magnification.

Lungs of HeV-infected AGMs were discolored, wet and heavy upon necropsy with areas of multifocal congestion and hemorrhage. Pathological findings were similar in NiV-infected AGMs (Figure 3). Infection of AGMs i.t. with NiV-M with doses ranging from 2.5 × 10^3^–1.3 × 10^6^ PFU resulted in mostly lethal disease with death occurring between 9- and 12-days following challenge [103]. Subjects demonstrated clinical signs of disease consistent with human NiV infection (acute respiratory distress, inappetence, lethargy, depression, fever, neurological signs) and NiV RNA was detectable in plasma samples as early as 7 dpi and in nasal swabs 4 dpi [103]. Notably, AGM models for both Malaysia and Bangladesh strains of NiV have been established [103] that are consistent with human disease [116,120]. In line with the high mortality rates in humans reported in outbreaks caused by the latter strain, NiV-B appears more pathogenic than NiV-M in the AGM model under identical experimental conditions, resulting in uniform lethality (NiV-B) as opposed to partial lethality (NiV-M) [116].

Severe neurological signs of disease, including seizure, headache, dizziness, myoclonus, and dysphasia, were reported in human NiV-M infections, with over 50% of patients presenting with reduced levels of consciousness and brain-stem dysfunction [23]. Notably, 78% of patients in the outbreak received either oral or intravenous ribavirin. Interestingly, treatment of HeV infection AGMs with ribavirin increased the propensity of animals to exhibit neurological signs (muscle fasciculations, seizure) and a delayed disease course (by 1–2 days) but ultimately did not improve survival [104]. Human patients infected with HeV have also received ribavirin therapy, and consistent with what was observed in AGMs, this treatment does not appear to benefit outcomes, although the small number of HeV patients prohibits definitive conclusions from being reached. One of the more promising treatments tested in NiV and HeV-infected AGMs is monoclonal antibodies targeting the surface glycoproteins of HNVs, including m102.4 [108,116]. Remdesivir (GS-5734) was also found to be protective [121]. A variety of vaccine countermeasures have been evaluated in the AGM model against NiV and HeV, including rVSV displaying NiV-B (G) glycoprotein [107,122], soluble HeV (G) glycoprotein [109,117], and a chimpanzee adenovirus platform (ChAdOx1) displaying the NiV-M (G) glycoprotein [123]. Thus, the AGM model is versatile and can be used to explore HNV pathogenesis (including neuropathologies) and to develop medical countermeasures.

### 8.2. Cynomolgus Macaque

Infection of cynomolgus macaques with 5 × 10^5^ PFU of HeV via the i.t./i.n. route caused very mild clinical disease in some animals with signs of agitation, increased aggressiveness, and abdominal breathing, accompanied by decreased white blood cell counts and increased levels of liver enzymes ALT and AST (Table 7) [105]. HeV RNA is detectable throughout the course of infection in cynomolgus macaques starting three days post-challenge in blood samples and oral, nasal, and rectal swabs, with levels similar to that of HeV-infected AGMs. No evidence of pathological lesions was observed at necropsy, nor was viral antigen detected. HeV-infected macaques seroconverted and achieved neutralizing antibody titers approximately equal to those seen to protect AGMs from lethal HNV challenge successfully. Assessment of global transcriptional changes related to immune signaling revealed that surviving HeV-infected macaques showed upregulated levels of transcripts mapped to adaptive immunity signaling pathways, including B-cell antigen presentation and differentiation into plasma cells. Instead, downregulated transcripts were implicated in complement activation, apoptosis, and cytotoxic granule release [105]. Additionally, macaques displayed lower levels of MCP-1, a chemoattractant involved in the trans-endothelial migration of monocytes, likely reducing the systemic spread of the virus following infection. Infection with 5 × 10^5^ PFU i.t./i.n. of NiV-M or NiV-B produced limited signs of clinical illness similar to what was observed following the HeV challenge. The lack of lethal infection, clinical disease, or obvious pathological lesions following challenge with HNVs indicates that the model is of little utility for the study of medical countermeasure efficacy against HNV infection; however, further study of the immune response could inform the development of immunomodulatory drugs.

### 8.3. Common Marmoset

The marmoset model is the most recent nonhuman primate model of HNV disease to be developed and recapitulates the respiratory features of human NiV infection (Table 7). Seven-year-old marmosets challenged via the i.t./i.n. route with 6.33 × 10^4^ PFU of NiV-B succumbed to disease 8–11 days after infection and showed clinical signs of anorexia, hyperventilation, hunched posture, and hind-limb tremors within eight days following challenge [106]. X-ray of the chest revealed opacities starting at day seven post exposure. Principal gross pathological findings included pulmonary edema and multifocal hemorrhage, with diffuse, macroscopic necrosis of the liver. Histopathological lesions included syncytial cells in the heart, kidney, and pulmonary vessels. Viral antigen was identified in the heart, pulmonary vessels, liver, kidney, and spleen; however, no lesions or antigens were found in the brain [106]. The marmoset model appears to replicate the respiratory pathology and systemic vasculitis caused by NiV infection in humans and AGMs, but the model lacks lesions in the brain typical of NiV encephalitis. One key advantage of the marmoset species as a model for HNV disease is their small size, which permits greater numbers of animals to be housed and included in studies, thereby increasing statistical power. However, the availability of immunological reagents may prohibit some studies from being performed, and the pathogenesis of HeV in this model has not been studied.

### 8.4. Squirrel Monkey

In a single study, squirrel monkeys were exposed to a NiV-M isolate using either intravenous (i.v.) or i.n. routes (Table 7) [124]. Both infection routes used viral doses of either 1 × 10^3^ or 1 × 10^7^ PFU. While clinical signs were observed in monkeys infected through both i.n. and i.v. routes, the duration of the disease was longer in i.n. infected animals (lasting seven days) compared to monkeys infected i.v. (lasting 2–3 days). Among i.v. infected monkeys, 3 out of 4 animals succumbed to the disease. Monkeys infected i.n. exhibited milder states of disease, with only 2 out of 4 animals showing signs before recovering after 3–7 days of illness [124]. The observed clinical indicators in these monkeys closely resembled those documented in human infections, with notable involvement of neurological and respiratory systems. NiV-specific RNA was detectable in various organs exclusively in monkeys infected i.v., accompanied by mild histologic lesions, primarily characterized by lung parenchymal inflammation. Unlike human infections, significant vasculitis and brain abnormalities were less pronounced. Nonetheless, immunohistochemistry demonstrated viral antigen localization in the brain, lungs, spleen, and kidney extravascular parenchyma, thus confirming viral presence in these organs [124].

## 9. Other Models of Note

HNVs emerged via zoonosis in livestock amplifying reservoirs horses (HeV) and pigs (NiV). Initial studies of HeV in horses were necessary to determine the etiology and characterize the causative agent of the outbreaks. Since horses and pigs can transmit HNVs to humans, the development of vaccine countermeasures to break transmission has been evaluated in these models. Other species, including cats and dogs, have been evaluated for HNV permissiveness [55]. The cat model was briefly pursued and used to evaluate an experimental vaccine for NiV infection [125], which proved effective. No further countermeasure testing was reported in this model. Similarly, only a single experimental challenge study in dogs has been performed to investigate the pathogenesis and transmission of HeV in this model. Dogs and cats are not consistently used as animal models for HNV disease for a variety of reasons, including their status as companion animals, their inability to fully recapitulate the HNV disease observed in humans, and the existence of better-suited species for biomedical research. Epidemiologic surveillance and observational studies of peridomestic animals, including cats and dogs, may be of use in outbreaks due to the potential for livestock to transmit HNVs to these species, which may then be capable of subsequent transmission to humans.

### 9.1. Horses

Natural HeV infection of horses leads to a severe and often fatal disease characterized by a sudden onset of influenza-like signs, including fever, inappetence, lethargy, respiratory signs of frothy nasal discharge, coughing, tachypnea, and neurological signs of aggression, muscle tremors, unsteadiness, head-tilt, and altered behavior [1]. Disease progression can be rapid, with horses deteriorating within a matter of days. Naturally, infected animals die or are typically humanely euthanized 5–15 days after showing clinical signs. Due to the severity of the disease and the potential for transmission to humans, HeV infection of horses is of significant concern for both equine and public health. Experimental infection of horses (Table 8) via i.v., i.n., or o.n. routes with HeV-containing tissue homogenate from fatal equine cases or cell culture isolates of HeV resulted in rapid and fatal disease characterized by severe pulmonary edema, congestion, focal hemorrhagic lesions and cyanosis of the lungs, and enlarged spleen [126]. Histopathological findings included the presence of syncytial cells in pulmonary vessels and the kidney, alveolar edema, and systemic vasculitis found in the brain, lungs, lymph nodes, kidney, spleen, uterus, and intestine [126,127]. Natural infection of horses with NiV has been reported [32]. A veterinary vaccine formulated with the soluble HeV-G glycoprotein is currently approved for use in Australia to protect horses from HeV infection and has demonstrated efficacy in preventing outbreaks [128].

### 9.2. Pigs

Experimental infection of pigs with NiV or HeV produces clinical disease with both respiratory and neurological features but typically does not result in fatal outcomes (Table 9). NiV infection of humans in Malaysia and Singapore in 1999 was preceded by widespread infection of pigs on farms, with animals typically displaying a characteristic barking cough and occasionally muscle spasms, myoclonus, trembling, and fever [129]. Although experimental infection often produces a mild, self-limiting disease with clinical signs evident by day 7 post-infection, NiV is shed in nasal secretions in pigs [130]. Gross macroscopic lesions are present in the lungs and include reddish-purple consolidation and hemorrhage. Histopathological lesions include syncytial cell formation, moderate to severe meningitis, and fibrinoid necrosis of pulmonary vessels [130,131]. Landrace pigs challenged with 6.6 × 10^7^ PFU of HeV via the o.n. route resulted in the development of clinical signs, including depression, inappetence, and weight loss as soon as four days post-infection [89]. Gottingen minipigs challenged with 2 × 10^7^ PFU i.n. developed clinical signs of the disease 3–7 days post-infection, and at necropsy, had evident petechial hemorrhage of the lungs and lymph nodes, with histological lesions including pulmonary edema and syncytial cells. Landrace pigs developed more severe interstitial pneumonia and extensive syncytial cell formation in the alveoli and bronchiolar epithelium; infiltration of inflammatory cells was observed in the bronchial lymph nodes [89]. A variety of veterinary vaccines have been experimentally evaluated in this model [131,132,133].

### 9.3. Dogs

Dogs were among the first species to be experimentally infected with HeV [55]. Several dogs have been observed to be directly infected with HeV or NiV, and serosurveillance suggests infections of dogs during NiV outbreaks are frequent. Livestock-to-dog and dog-to-human transmission events pose a potential transmission risk during outbreaks. Dogs are not a natural host of HeV or NiV but potentially serve as amplifying reservoirs during epidemics. Infection of dogs with Hendra virus has been described in two experimental challenge studies (Table 10) and a case report of natural HeV infection [138], which generally describe subclinical illness with limited evidence of pathologies in the respiratory or central nervous system.

HeV is not highly pathogenic in canines, but viremia and oral shedding suggest dogs are capable of transmitting the virus to humans. The challenge of dogs with 5 × 10^3^ TCID_50_ of the prototype HeV strain via the s.c. route produced no clinical signs of disease, animals did not seroconvert, and gross or histopathological lesions were absent upon necropsy [55]. In 2013, a dog was found to be naturally infected with HeV following contact with an HeV-infected horse during an outbreak and was subsequently euthanized following confirmation of infection [138]. The dog did not show clinical signs of illness, but gross- and histopathological lesions were discovered at necropsy. Primary gross lesions included reddening of the lungs, frothy edematous fluid in the trachea and bronchi, and hyperplasia of mandibular, tonsillar, bronchial, and mediastinal lymph nodes. Significant vasculitis and fibrinoid necrosis were identified in the lymph nodes, kidneys, and brain, in addition to the spleen, lungs, intestines, and liver [138]. Further histopathological lesions were identified throughout the brain, including the brain stem and meninges. Although the virus was not isolated from tissues, the dog was seropositive and viral antigen was present in the kidney [138].

Experimental challenge of beagles with HeV dosed at 2 × 10^6^ TCID_50_ via the o.n. route was not lethal and produced only mild clinical signs of disease, including inappetence and low fever at days 6 and 10 post-infection [139]. The virus was re-isolated from oral swabs at days 2 and 4 post-infection and from respiratory tissues at days 2, 4, 6, and 8; however, it was not recovered from the blood at any point [139]. Gross pathological lesions at necropsy were generally consistent with those observed in natural infection and included mild consolidation of the lungs, hyperplasia of the tonsils and tracheobronchial lymph nodes, and areas of focal hemorrhage and congestion of the mediastinal and retropharyngeal lymph nodes [138,139]. Histopathological lesions of the lungs included bronchointerstitial pneumonia, vasculitis, and antigen deposition in the bronchiolar epithelium and vascular endothelium. Limited syncytial cell formation was observed in the bronchiole lymph nodes. Notably, no evidence of neuroinfection from clinical signs or histopathological lesions was observed in experimentally infected dogs [138,139]. These findings suggest that experimental and natural HNV infections produce mild disease in dogs. Oral swabs collected from HeV-infected dogs at days 2 and 4 following the challenge were used to inoculate naïve ferrets. Ferrets developed fever, depression, and paralysis and were euthanized 7–9 days after infection, demonstrating that virus shedding from HeV-infected dogs was infective to naïve, susceptible animals [139]. This finding suggests that dogs may potentially pose a risk to humans, horses, and other peridomestic animals during HeV outbreaks.

During the 1998–1999 outbreak of NiV in Malaysia, it was reported that domestic dogs were also infected [140]. Serosurveillance in April of 1999 identified that 57% of 63 dogs sampled were positive for NiV-reactive antibodies, and additional measurements from the same area in May of 1999 saw a non-significant decrease in percent positivity to 26%, suggesting that dog-to-dog transmission was not taking place [141]. Limited data is available for dogs confirmed to have been actively infected with NiV, and no studies involving experimental infection of dogs with NiV have been published to date. During the 1999 outbreak, one moribund and one deceased dog were necropsied, with clinical signs observed in the moribund dog being respiratory distress, fever, nasal discharge, and conjunctivitis [142]. Gross findings at necropsy were consistent with human respiratory pathologies: heavy lungs with visible congestion, mottling, and consolidation of all lobes in addition to reddening of the renal capsules and cortices [142]. Histological lesions were reported in the lungs (severe pulmonary edema, interstitial pneumonia), kidneys (glomerular atrophy and syncytia), and brain (nonsuppurative meningitis). Virus isolation or antigen deposition in tissues was not described [142]. This report and evidence of seroprevalence during active NiV outbreaks indicate that close attention should be paid to dogs during outbreaks. Furthermore, disease control practices should be enforced to reduce and prevent contact between dogs and known amplifying reservoirs of NiV. Further epidemiological surveillance and investigation of apparent NiV- or HeV-infected animals during outbreaks should be a priority to understand modes of HNV transmission further.

### 9.4. Cat

Experimental challenge of cats revealed high susceptibility to both HeV and NiV by a variety of challenge routes and doses (Table 11). The challenge of cats with ~1 × 10^3^–5 × 10^4^ TCID_50_ of HeV s.c., o.n., or oral (o.r.) routes produces influenza-like clinical signs characterized by fever and respiratory distress [55,143]. Gross pathology in HeV-infected felines was characteristic of acute HeV disease with focal hemorrhagic lesions of the lungs, pulmonary edema and hydrothorax, and the enlargement of the spleen and mesenteric lymph nodes [86]. Histopathologic lesions were consistent with those observed in horses, including systemic vasculitis with syncytial cells, fibrinoid necrosis, and mononuclear inflammatory cell infiltration in the lungs. The virus was isolated from the respiratory tract, liver, spleen, kidney, lymph node, bladder, pleural fluid, urine, and brain. Neurological signs were not reported [86,143].

Infection of domestic shorthair cats with 5 × 10^2^–5 × 10^3^ TCID_50_ of NiV-M via the s.c. or o.n. route resulted in clinical disease characterized by fever, depression, and respiratory distress. NiV infection produced lesions highly similar to HeV-infected cats, with focal hemorrhagic necrosis of the pleura and wet and heavy lungs with edema and hydrothorax. Histopathological lesions of the lungs included bronchitis and alveolar hemorrhage. Vasculitis with syncytial cell formation and inflammatory cell infiltrates was observed in large arterial vessels of the lungs and endothelial cells of the lungs, spleen, lymph nodes, and brain [125,130]. Meningitis was also found. NiV antigen was detected in the lungs, spleen, bladder, and lymph nodes, where the infectious virus was recoverable, in addition to the tonsil [125,130]. Transmission of NiV to or from cats has not been documented during outbreaks; however, vertical transmission of NiV was observed in a cat discovered to be pregnant at necropsy [145], with infectious virus found in the placenta, fetus and uterine fluid.

## 10. Future Directions

Well-characterized animal models for both NiV and HeV are currently required for investigating the efficacy of medical countermeasures against these pathogens. Here, we describe several highly reliable models for recapitulating human infection by HNVs. AGMs closely mimic the pathogenesis and clinical signs of NiV and HeV disease in humans and are the gold standard for evaluating medical countermeasures, including vaccines, antivirals, and monoclonal antibodies. Hamsters and ferrets are the most well-characterized small- and mid-sized animal models for HNV disease, and continued optimization and refinement of these models has allowed for pathogenesis and countermeasure efficacy studies for HeV and NiV. Continued development of these models should be pursued to allow for better harmonization of challenge dosing and clinical scoring. The field should also prioritize the development and validation of species-specific reagents for hamsters and ferrets, as the lack of commercial availability fetters opportunities for more precise characterization of HNV pathogenesis. Models consistently recapitulating the CNS pathologies of HNV infection would also prove useful, especially for studying the recrudescence and chronic encephalitis seen in human NiV infection. This would allow for the evaluation of CNS-based therapeutics for HNV disease.

The seasonal nature of NiV and HeV virus outbreaks in their respective endemic areas continues to pose a risk to public health. The frequency and severity of NiV outbreaks in Bangladesh and India appear to be increasing, with a recent outbreak resulting in 11 cases and eight deaths earlier this year. The wide geographic distribution of natural reservoirs of HNVs and the emergence of novel HNVs, including Cedar, Mojiang and Langya, suggest continued efforts are needed to understand these viruses. Animal models have been pivotal in our study of HeV and NiV disease pathogenesis and for evaluating medical countermeasures to potentially protect against future outbreaks of these deadly viruses.

## Figures and Tables

**Table 1:** Experimental findings in immunocompetent mouse models of HNV Disease.

Species (Age)	Virus (Isolate)	Dose	Route	% Lethality	Clinical Disease	Reference
C57BL/6 (aged)	HeV (Redlands/Horse/2008)	5 × 10^4^ TCID_50_	i.n.	100% (5/5)	Depression, ataxia, hypersensitivity, tremors	[56]
BALB/c (aged)				60% (3/5)		
C57BL/6 (juvenile)			s.c.	None	None	
BALB/c (juvenile)						
C57BL/6 (aged)	NiV-B (human/2004/Rajbari, R1)	5 × 10^4^ TCID_50_	i.n.	None	None	[57]
C57BL/6 (juvenile)						
BALB/c (aged)						
BALB/c (juvenile)						
C57BL/6 (aged)	NiV-M (Malaysia/human/99)	5 × 10^4^ TCID_50_	i.n.	None	None	
C57BL/6 (juvenile)						
BALB/c (aged)						
BALB/c (juvenile)						
Unknown	HeV (prototype)	5 × 10^3^ TCID_50_	s.c.	None	None	[55]
Swiss (juvenile)	HeV (prototype)	6 × 10^5^ PFU	i.n.	None	None	[43]
		1 × 10^7^ PFU	i.p.			
C57BL/6	NiV-M (UMMC1)	1 × 10^6^ PFU	i.p.	None	None	[58]
		1 × 10^5^ PFU	i.c.	100% (5/5)	Weight loss, aggressiveness, prostration, paralysis	[58]
Suckling Mice (2–3-day-old)	NiV-M	1 × 10^0^ TCID_50_	i.c.	15% (9–13) ^a^	Clinical signs including weight loss ^b^	[59]
		1 × 10^1^ TCID_50_		66% (9–13) ^a^		
		1 × 10^2^ TCID_50_		100% (9–13) ^a^		
		1 × 10^3^ TCID_50_		100% (9–13) ^a^		
		1 × 10^4^ TCID_50_		100% (9–13) ^a^		
		1 × 10^5^ TCID_50_		100% (9–13) ^a^		
		1 × 10^6^ TCID_50_		100% (9–13) ^a^		
BALB/c (13-week-old)	NiV-B (human/2004/Rajbari, R1)	5 × 10^4^ TCID_50_	i.n.	None	None	[60]
	rNiV-WT			None		
	rNiV-F_HeV_			None		
	rNiV-G_HeV_			None		
	rNiV-M_HeV_			None		
	HeV (Redlands/Horse/2008)	5 × 10^4^ TCID_50_	i.n.	None	None	
	rHeV-WT			40% (2/5)	Depression (2/5), increased respiration (2/5), hypersensitivity to stimuli (2/5), piloerection (1/5), hunched posture (1/5)	
	rHeV-F_NiV_			None	None	
	rHeV-G_NiV_			None		
	rHeV-M_NiV_			None		

^a^ A range of 9–13 animals per group. ^b^ Comprehensive numerical clinical scores were described in this study, with individual signs not specified for subjects.

**Table 3:** Experimental findings in Syrian golden hamster models of HNV Disease.

Age	Virus (Isolate)	Dose	Route	% Lethality	Clinical Disease	Reference
8–10-week-old	NiV-M	1 × 10^4^ TCID_50_	i.p.	80% (4/5)	Weight loss	[68]
	HeV (prototype)	1 × 10^4^ TCID_50_	i.p.	100% (5/5)	Head tilt, weight loss, hypothermia,	
7-week-old	HeV (prototype)	1 × 10^5^ PFU	i.p.	100% (6/6)	Paralysis, trembling limbs, breathing difficulties, serosanguinous nasal discharge	[65]
		1 × 10^4^ PFU		100% (6/6)		
		1 × 10^3^ PFU		100% (6/6)		
		1 × 10^2^ PFU		83% (5/6)		
		1 × 10^1^ PFU		67% (4/6)		
		1 × 10^0^ PFU		None	None	
11-week-old		1.2 × 10^3^ PFU		80% (4/5)	Paralysis, trembling limbs, breathing difficulties, serosanguinous nasal discharge	
7-week-old		1.2 × 10^3^ PFU		100% (5/5)		
11-week-old		1.2 × 10^4^ PFU		100% (5/5)		
7-week-old		1.2 × 10^4^ PFU		100% (5/5)		
2-month-old	NiV-M	6 × 10^5^ PFU	i.n.	100% (2/2)	Ataxia, imbalance	[43]
		1 × 10^7^ PFU	i.p.	100% (2/2)		
7–14-week-old		1 × 10^4^ PFU	i.p.	100% (6/6)	Tremors, limb paralysis	
		1 × 10^3^ PFU		83% (5/6)		
		1 × 10^2^ PFU		67% (4/6)		
		1 × 10^1^ PFU		None (0/6)		
		1 × 10^0^ PFU		None (0/6)		
7–14-week-old		1 × 10^6^ PFU	i.n.	83% (5/6)	Imbalance, limb paralysis, lethargy, muscle twitching, breathing difficulties	
		1 × 10^5^ PFU		83% (5/6)		
		1 × 10^4^ PFU		50% (3/6)		
		1 × 10^3^ PFU		50% (3/6)		
		1 × 10^2^ PFU		None (0/6)		
		1 × 10^1^ PFU		None (0/6)		
~10-week-old ^a^	NiV-M	6.84 × 10^4^ TCID_50_	i.p.	100% (10/10)	Severe clinical signs of disease, not described	[71]
~10-week-old	NiV-M	1 × 10^5^ TCID_50_	i.p.	100% (3/3)	Neurological signs, respiratory distress	[75]
8-week-old	NiV (unknown)	2 × 10^3^ TCID_50_	i.p.	90% (9/10)	Not described	[76]
Unknown	NiV-M ^b^	1 × 10^3^ PFU	i.p.	100% (unknown)	Not described	[74]
6–8-week-old	NiV-M	1 × 10^7^ TCID_50_	i.n.	100% (14/14) ^c^	Severe signs of disease, respiratory distress	[77]
		1 × 10^5^ TCID_50_		67% (4/6)	Weight loss	
		1 × 10^3^ TCID_50_		33% (2/6)	Weight loss, neurological signs	
6-week-old	NiV-M	1 × 10^5^ TCID_50_	i.n.	100% (5/5)	Weight loss, severe acute respiratory distress	[78]
		1 × 10^2^ TCID_50_		100% (5/5)	Weight loss, neurological signs (complete paralysis, seizure, muscle fasciculations)	
	HeV (prototype)	1 × 10^5^ TCID_50_	i.n.	100% (5/5)	Weight loss, severe acute respiratory distress (labored breathing, serosanguineous oronasal exudates)	
		1 × 10^2^ TCID_50_		100% (5/5)	Weight loss, respiratory disease, neurological signs (complete paralysis, seizure, muscle fasciculations)	
5–6-week-old	NiV-M	1 × 10^5^ TCID_50_	i.p.	100% (5/5)	Respiratory distress (labored breathing, hunched posture), neurological signs (one animal)	[79]
		1 × 10^4^ TCID_50_		100% (5/5)	Respiratory distress (labored breathing, hunched posture)	
		1 × 10^3^ TCID_50_		100% (5/5)	Neurological signs (imbalance, partial paralysis, seizure)	
		1 × 10^2^ TCID_50_		60% (3/5)	Neurological signs (imbalance, partial paralysis, seizure)	
		1 × 10^1^ TCID_50_		None (0/5)	Neurological signs	
		1 × 10^0^ TCID_50_		None (0/5)		
5–6-week-old	NiV-B	1 × 10^5^ TCID_50_	i.p.	80% (4/5)	Respiratory or neurological, both respiratory and neurological (one animal)	
		1 × 10^4^ TCID_50_		80% (4/5)	Respiratory or neurological, both respiratory and neurological (one animal)	
		1 × 10^3^ TCID_50_		60% (5/5)	Neurological signs	
		1 × 10^2^ TCID_50_		None (0/5)		
		1 × 10^1^ TCID_50_		40% (2/5)		
		1 × 10^0^ TCID_50_		None (0/5)		
5–6-week-old	NiV-M	1 × 10^5^ TCID_50_	i.n.	100% (5/5)	Not described	
5–6-week-old	NiV-B	1 × 10^5^ TCID_50_		100% (5/5)		
4–5-week-old	NiV-M	1 × 10^5^ PFU	aerosol	100% (5/5)	Weight loss, scruffy coat (3/5), Respiratory and neurological signs (2/5)	[80]
		2 × 10^4^ TCID_50_		60% (3/5)	Neurological signs (3/5), Respiratory and neurological signs (2/5)	
		8 × 10^2^ TCID_50_		80% (4/5)	Neurological signs (3/5), Respiratory and neurological signs (2/5)	
		2.5 × 10^2^ TCID_50_		80% (4/5)	Neurological signs (3/5), Respiratory and neurological signs (2/5)	
4–5-week-old	rNiV-Fluc^NP^	2 × 10^5^ PFU	aerosol	100% (5/5)	Weight loss, scruffy coat, labored breathing (4/5), Respiratory and neurological symptoms (1/5)	
		8.5 × 10^4^ TCID_50_		100% (5/5)	Respiratory and neurological signs (4/5), weight loss	
		9.5 × 10^3^ TCID_50_		100% (5/5)	Respiratory and neurological signs (4/5), weight loss, weight loss (1/5), respiratory only (1/5)	
		2.5 × 10^2^ TCID_50_		60% (3/5)	Respiratory and neurological signs (2/5), weight loss, respiratory only (1/5)	
		6.6 × 10^1^ TCID_50_		60% (3/5)	Respiratory and neurological signs (2/5), weight loss, respiratory only (1/5)	
Unknown	NiV-M (UMMC1) ^d^	1 × 10^4^ PFU	i.p.	100% (6/6)	Weight loss, dyspnea, tremor, paralysis	[81]
Unknown	HeV (prototype) ^d^	1 × 10^4^ PFU		100% (6/6)	Weight loss, dyspnea, tremor, paralysis	
8-week-old	rNiV(W-) ^e^	1 × 10^5^ PFU	i.p.	83% (5/6)	Neurological signs	[82]
		1 × 10^4^ PFU		83% (5/6)		
		1 × 10^3^ PFU		66% (4/6)		
		1 × 10^1^ PFU		50% (3/6)		
		1 × 10^0^ PFU		16% (1/6)		
6–11-week-old	NiV-M	1 × 10^5^ TCID_50_	i.n.	100% (10/10)	Respiratory signs (dyspnea, wasp waist), ataxia (2/10)	[83]
		1 × 10^4^ TCID_50_		100% (4/4)	Dyspnea (1/4), ataxia (2/4)	
	NiV-M	1 × 10^3^ TCID_50_	i.p.	100% (8/8)	Neurological signs, ataxia (2/8), respiratory signs (2/8).	
		1 × 10^2^ TCID_50_		75% (3/4)	Neurological signs	
8-week-old	NiV-M (UMMC1)	1 × 10^2^ PFU	i.p.	100% Unknown ^f^	Not described	[84]
		1 × 10^3^ PFU		100% Unknown ^f^		
	NiVΔC1 ^g^	1 × 10^2^ PFU	i.p.	~30–40% Unknown ^f^	Not described	
		1 × 10^3^ PFU		~80–90% Unknown ^f^		
	NiVΔC2 ^g^	1 × 10^2^ PFU	i.p.	~60% Unknown ^f^	Not described	
		1 × 10^3^ PFU		~70% Unknown ^f^		
5–7- week-old	NiV-M	1 × 10^6^ TCID_50_	i.n.	76% (~35/46) ^h^	Clinical signs including weight loss ^i^	[59]
		1 × 10^5^ TCID_50_		50% Unknown		
		1 × 10^3^ TCID_50_		12% Unknown		
		1 × 10^4^ TCID_50_	i.p.	100% Unknown		

^a^ Age at the time of challenge. ^b^ Received a nonspecific vaccinia vaccine. ^c^ Animals from two control groups combined. ^d^ Received a nonspecific AAV-GFP vaccine. ^e^ Recombinant NiV with W accessory protein knockout. ^f^ Study specifies 5 or 6 animals per group. ^g^ Recombinant NiV mutants lacking C protein expression. ^h^ Includes historical data. ^i^ Comprehensive numerical clinical scores were described in this study, with individual signs not specified for subjects.

**Table 4:** Experimental findings in guinea pig models of HNV Disease.

Virus (Isolate)	Dose	Route	% Lethality	Clinical Disease	Reference
HeV (prototype)	5 × 10^3^ TCID_50_	s.c.	80% (4/5)	Respiratory distress, inappetence	[55]
	5 × 10^4^ TCID_50_	s.c.	75% (9/12)	Weakness, lethargy, head tilt, depression, abortion (1/12)	[88]
	3.4 × 10^6^ PFU	i.n.	33% (2/6)	Depression, inappetence, inactivity, weight loss, nasal hemorrhage (1/6)	[89]
NiV-M	6 × 10^5^ PFU	i.n.	None	None	[43]
	1 × 10^7^ PFU	i.p.	None	Transient fever and weight loss	
	5 × 10^4^ TCID_50_	i.p.	38% (3/8)	Ruffled fur, ataxia (1/8)	[90]
	6 × 10^4^ PFU	i.p.	92% (24/26)	Severe clinical disease (features not described)	[91]

**Table 5:** Experimental findings in ferret models of HNV Disease.

Virus (Isolate)	Dose	Route	Treatment	% Lethality	Clinical Disease	Reference
NiV-M (EUKK 19817)	5 × 10^4^ TCID_50_	o.n.	N/A	100% (2/2)	Severe depression, orthopnea, expiratory dyspnea, serous nasal discharge, cough, subcutaneous edema of the head	[92]
	5 × 10^3^ TCID_50_			100% (2/2)	Severe depression, orthopnea, cutaneous ecchymoses, vomiting, hypothermia	
	5 × 10^2^ TCID_50_			50% (1/2)	Obtundation, tremor, and hind limb paralysis	
	5 × 10^1^ TCID_50_			None (0/2)	None	
	5 × 10^3^ TCID_50_		PBS (IV)	100% (2/2)	Weight loss, fever, depression, reduced play activity, subcutaneous edema of the head, cutaneous hemorrhages, inappetence (1/2), diarrhea (1/2), blood in the mouth (1/2)	
			mAb ^a^ (IV)	66% (2/3)	Fever, depression, weight loss, reduced play activity, inappetence, diarrhea, crusting nasal discharge, hind limb paralysis (1/2), generalized tremor (1/3)	
HeV (Redlands 2008)	5 × 10^4^ TCID_50_	o.n.	N/A	100% (2/2)	Fever, depression, decreased grooming, generalized tremors	[93]
	5 × 10^3^ TCID_50_			100% (2/2)		
	5 × 10^2^ TCID_50_			100% (2/2)		
	5 × 10^1^ TCID_50_			100% (2/2)		
	5 × 10^3^ TCID_50_		HeVsG 100 μg ^b^	None (0/2)	None	
			HeVsG 20 μg ^b^	None (0/2)		
			HeVsG 4 μg ^b^	50% (1/2)	Reduced play activity, weakness, tremor	
			CpG Adjuvant ^c^	100% (2/2)	Reduced play activity, fever, hind limb paralysis	
NiV-B (human/2004/Rajbari, R1)	5 × 10^3^ TCID_50_	unknown	HeVsG 100 μg ^b,d^	None (1/1)	None	[94]
			HeVsG 20 μg ^b,d^	None (2/2)		
			HeVsG 4 μg ^b,d^	None (1/1)		
			HeVsG 100 μg ^b,e^	None (1/1)		
			HeVsG 20 μg ^b,e^	None (1/1)		
			HeVsG 4 μg ^b,e^	None (2/2)		
			CpG Adjuvant ^c^	100% (2/2)	Fever	
			CpG Adjuvant ^c^	100% (2/2)	Fever, reduced playfulness	
NiV-M (patient isolate 1998 Malaysia outbreak)	1 × 10^5^ TCID_50_	i.n.	N/A	100% (4/4)	Labored breathing, fever, mild paralysis, generalized tremors, subcutaneous edema of head and neck, lack of grooming, hunched posture, ataxia, continuous licking, imbalance, myoclonus, head tilt, hind-limb paralysis, seizures	[95]
	1 × 10^3^ TCID_50_			100% (4/4)		
	1 × 10^2^ TCID_50_			100% (4/4)		
	1 × 10^1^ TCID_50_			25% (1/4)		
NiV-B (2004 patient isolate)	1 × 10^5^ TCID_50_	i.n.	N/A	100% (4/4)		
	1 × 10^3^ TCID_50_			100% (4/4)		
	1 × 10^2^ TCID_50_			75% (3/4)		
	1 × 10^1^ TCID_50_			25% (1/4)		
HeV (prototype)	1 × 10^5^ TCID_50_	i.n.	N/A	100% (4/4)		
	1 × 10^3^ TCID_50_			100% (4/4)		
	1 × 10^2^ TCID_50_			100% (4/4)		
	1 × 10^1^ TCID_50_			75% (3/4)		
NiV-M (isolate 1999011924)	5 × 10^3^ PFU	i.n.	N/A	100% (1/1)	Fever, facial edema, nasal and ocular discharge, sneezing, depression, loss of appetite, labored breathing, head and neck myoclonus	[96]
			mAb ^f^	None (0/3)	Fever (1/3)	
			mAb ^g^	None (0/3)	Fever (1/3), minor facial and ear twitching (1/3)	
HeV (prototype)	5 × 10^3^ PFU	i.n.	N/A	100% (1/1)	Fever, nasal and ocular discharge, sneezing, loss of appetite, depression, facial edema, labored breathing	
			mAb ^g^	None (0/3)	Mild fever	
NiV-B (human/2004/Rajbari, R1)	5 × 10^3^ PFU	o.n.	N/A	100% (4/4)	Fever, ataxia, agitation, facial edema, disorientation, tachypnea/dyspnea	[97]
		contact ^h^		100% (4/4)		
NiV-M (Malaysia/Human/99)	5 × 10^3^ PFU	o.n.	N/A	100% (4/4)		
		contact ^h^		100% (4/4)		
NiV-B (human/2004/Rajbari, R1)	5 × 10^3^ TCID_50_	o.n.	N/A	100% (8/8)	Hunched posture, agitation, sneezing, weight loss, licking, dehydration, vomiting, myoclonus of forelimbs, hindlimbs, flank, or tail, nasal discharge, facial edema, oral mucosa hemorrhage, ataxia, ventral neck edema, paralysis	[98]
NiV-M (Malaysia/Human/99)	5 × 10^3^ TCID_50_	o.n.	N/A	100% (6/6) ^i^	Severe ataxia, facial and hind limb tremors, head tilt, torticollis, sneezing, nasal discharge, facial edema, hemorrhage of rectal mucosa, dyspnea, hemorrhage from nose and mouth, spastic paralysis of right forelimb, myoclonus of right trunk, extensive cutaneous petechial hemorrhage, hind limb paralysis, hunched posture, muscular fasciculation over flank, weight loss, recumbency	
NiV-M (EUKK 19817)	5 × 10^3^ TCID_50_	o.n.	Vehicle control ^j^	100% (2/2)	Fever, depression, hind-limb paralysis, myoclonus, urinary incontinence, subcutaneous edema of neck and throat, cutaneous petechial hemorrhage, serosanguinous oral secretions	[99]
			Antiviral ^k^	100% (3/3)		
			Antiviral ^l^	100% (3/3)		
rNiV_M_-wt	5 × 10^3^ PFU	i.n.	N/A	100% (5/5)	Fever, respiratory distress, lethargy, inappetence, depression, ocular, oral, and nasal discharge, ataxia, severe hypothermia, myoclonus, weight loss, hindlimb paresis, rales, hyperglycemia	[100]
rNiV_M_-P_Y166E_	5 × 10^3^ PFU	i.n.	N/A	100% (5/5)	Fever, depression, lethargy, inappetence, oral and nasal discharge, seizure, weight loss, aggressiveness, hyperglycemia, ataxia, hindlimb paresis	
rNiV_M_-P_D116–135_	5 × 10^3^ PFU	i.n.	N/A	100% (5/5)	Fever, depression, lethargy, inappetence, sneezing, myoclonus, facial tremor, nasal discharge, weight loss, hindlimb paresis, quadriparesis, hypothermia, seizure	
rNiV_M_-wt	5 × 10^3^ PFU	i.n.	N/A	100% (5/5)	Depression, lethargy, inappetence, ocular and nasal discharge, myoclonus, ataxia, weight loss, sneezing, obtundation, nasal and oral frothing, facial edema, tremor	[101]
rNiV_M_-W^KO^	5 × 10^3^ PFU	i.n.	N/A	100% (5/5)	Depression, lethargy, dehydration, sneezing, rales, nasal and oral frothing, severe ataxia, seizure, hypothermia, inappetence, hypersalivation, tremors, ocular and nasal discharge, obtundation	
rNiV_M_-V^KO^	5 × 10^3^ PFU	i.n.	N/A	None (0/5)	Lethargy, nasal discharge, depression, tremors, mild ataxia	
rNiV_M_-wt	5 × 10^3^ PFU	i.n.	N/A	100% (5/5)	Depression, lethargy, inappetence, ocular and nasal discharge, ataxia, hindlimb myoclonus, tremors, nasal and oral frothing, facial edema, rales, hypothermia, sneezing, weight loss, obtundation	[102]
rNiV_M_-C^KO^	5 × 10^3^ PFU	i.n.	N/A	100% (5/5)	Depression, lethargy, sneezing, ataxia, hypothermia, inappetence, myoclonus, rales, ocular and nasal discharge, weight loss	
rNiV_M_- C^KO^W^KO^	5 × 10^3^ PFU	i.n.	N/A	60% (3/5)	Depression, lethargy, sneezing, nasal discharge, rales, hindlimb myoclonus and paralysis, severe tremors, seizures, weight loss, facial myoclonus, dilated pupils, facial edema, obtundation, severe hypothermia, visual deficit, aggressiveness	

^a^ mAb m102.4, 50 mg per animal, given 24 h prior to infection with NiV-M. ^b^ HeVsG is an adjuvanted, soluble HeV (G) glycoprotein vaccine containing a dose indicated in micrograms with a CpG adjuvant. ^c^ Ferrets received 150 μg CPG ODN 2007 dinucleotide adjuvant. ^d^ Vaccinated 20 days prior to challenge. ^e^ Vaccinated 434 days prior to challenge. ^f^ Received mAb h5B3.1, 20 mg/kg intraperitoneally on days 1 and 3 post-challenge. ^g^ Received mAb h5B3.1, 20 mg/kg intraperitoneally on days 3 and 5 post-challenge. ^h^ Ferrets were infected through cohabitation with directly exposed ferrets and inoculation with oronasal secretions of directly exposed ferrets at two time points. ^i^ Excluded one animal from the group due to euthanasia for reasons unrelated to NiV-M infection. ^j^ 20% sucrose. ^k^ Treated with chloroquine 24 h before challenge. ^l^ Treated with chloroquine 10 h after challenge.

**Table 6:** Experimental findings in AGM models of HNV Disease.

Virus (Isolate)	Dose	Route	% Lethality	Clinical Disease	Reference
HeV (prototype)	4 × 10^5^ TCID_50_	i.t.	100% (4/4)	Fever, severe respiratory distress (increased breathing rate, open mouth breathing), interstitial pneumonia (radiology)	[108]
	5 × 10^5^ PFU		100% (4/4)	Fever, depression, lethargy, inappetence, labored breathing, hind limb paralysis (1/4), dehydration	[109]
	4 × 10^5^ TCID_50_		100% (3/3)	Nasal discharge, labored breathing	[104]
HeV (prototype)	4 × 10^5^ TCID_50_	i.t.	100% (9/9) ^a^	Nasal discharge, labored breathing, seizure (1/9), muscle fasciculations (5/9)	
NiV-M	1.3 × 10^6^ PFU	i.t., oral	100% (1/1)	Depression, lethargy, fever, inappetence, severe dyspnea, labored breathing	[103]
	7.0 × 10^3^ PFU		None (0/1)	Depression, lethargy, fever, inappetence, severe dyspnea, labored breathing, nausea, lymphadenopathy, ecchymotic rash at the venipuncture site, muscle twitches, behavioral changes	
	8.1 × 10^4^ PFU		100% (1/1)	Depression, lethargy, fever, inappetence, severe dyspnea, labored breathing, nausea, pleural effusions (X-ray)	
	6.5 × 10^4^ PFU	i.t.	100% (1/1)	Depression, lethargy, fever, inappetence, labored breathing, pleural effusions (X-ray)	
	5.9 × 10^4^ PFU		100% (1/1)	Depression, lethargy, inappetence, labored breathing, X-ray showed pleural effusions	
	2.3 × 10^4^ PFU		100% (1/1)	Depression, lethargy, fever, inappetence, labored breathing, loss of balance, pleural effusions (X-ray)	
	7.0 × 10^3^ PFU		100% (1/1)	Depression, lethargy, inappetence, labored breathing, pleural effusions (X-ray)	
	2.5 × 10^3^ PFU		100% (1/1)	Depression, lethargy, inappetence, labored breathing, pleural effusions (X-ray)	
NiV-B (isolate 200401066)	8.81 × 10^2^ PFU	aerosol	100% (1/1)	Anorexia	[110]
	1.33 × 10^3^ PFU		100% (1/1)	Depression, lethargy, recumbency, anorexia, severe dyspnea, hypothermia	
	9.95 × 10^3^ PFU		100% (1/1)	Depression, lethargy, anorexia, fever, severe dyspnea, pulmonary consolidation (X-ray)	
	1.31 × 10^4^ PFU		100% (1/1)	Anorexia, mild dyspnea	
NiV-M	~64 × 10^1^ PFU ^b^	aerosol	100% (3/3)	Weight loss, cough, lethargy, inappetence, pulmonary consolidation (CT scan), neutrophilia (2/6), anemia (2/6), monocytosis (2/6), lymphopenia (3/6), hypoalbuminemia, increased globulin, increased respiratory rate only on day of euthanasia	[111,112]
	~7 × 10^2^ PFU ^b^		66% (2/3)		
NiV-M	2.41 × 10^5^ PFU	aerosol	100% (4/4)	Decreased activity, labored breathing, increased respiratory rates, unresponsiveness, fever, continuous head twitch (1/4)	[113]
NiV-M	2.5 × 10^4^ PFU	i.t.	50% (2/4)	Decreased responsiveness, tachypnea, fever, tachycardia, tachypnea (3/4), hypotension (2/4), hypothermia (2/4), tremors (1/4), bloody oronasal exudate (1/4), lymphadenopathy, weight loss (1/4)	[114]
NiV-M	1.3 × 10^4^ PFU	i.t.	100% (3/3)	Lethargy, cough, breathing difficulty, inappetence	[115]
	4.03 × 10^4^ PFU	aerosol	100% (3/3)		
NiV-M (isolate 199902916)	5 × 10^5^ PFU	i.t./i.n.	50% (2/4)	Depression, lethargy (3/4), fever, inappetence, dyspnea, lymphopenia, thrombocytopenia, hypoalbuminemia, nasal exudate (3/4), tremors (2/4)	[116]
NiV-B (isolate 200401066)	5 × 10^5^ PFU	i.t./i.n.	100% (4/4)	Fever (2/4), depression, inappetence, severe dyspnea, lymphopenia, thrombocytopenia, hypoalbuminemia, labored breathing (3/4), nasal exudate (2/4), with epistaxis (1/4), tremors (1/4)	
			100% (2/2) ^c^	Fever, depression, lethargy, inappetence, severe dyspnea, labored breathing, nasal exudate with epistaxis (1/2)	
NiV-B (isolate 200401066)	5 × 10^5^ PFU	i.t./i.n.	100% (6/6)	Aggressiveness, pulmonary edema (3/6), abdominal (3/6) or open mouth breathing (2/6), lethargy (4/6), depression (1/6), dyspnea (1/6), anorexia (3/6), recumbency (3/6), bradypnea (2/6), nasal exudate (1/6), hypothermia (1/6)	[105]
NiV-M (isolate 199902916)	5 × 10^5^ PFU	i.t./i.n.	50% (1/2)	Depression, abdominal breathing, anorexia (1/2), lethargy (1/2), recumbency (1/2), nasal exudates, pulmonary consolidation (x-ray, 1/2), tremors (1/2)	
HeV-prototype (isolate 9409-30-1800)	5 × 10^5^ PFU	i.t./i.n.	80% (4/5)	Anorexia (2/5), abdominal breathing (2/5), severe bradypnea (1/5), lethargy (1/5), dyspnea (1/5), mild tachypnea (1/5), pulmonary edema (1/5), depression (1/5)	
NiV-M	1 × 10^5^ TCID_50_	i.t.	100% (1/1) ^d^	Anorexia, depression, decreased activity, hunched posture, increase in respiratory rate, acute respiratory distress, decrease in platelet count	[117]
NiV-B (isolate 200401066)	5 × 10^5^ PFU	i.t./i.n.	100% (6/6) ^e^	Increased respiration rates, tachypnea, dyspnea, depression	[118]
NiV (unknown isolate/strain)	1 × 10^8^ TCID_50_	i.n./oral	None (0/1)	Weight loss, severe illness	[76]
	1 × 10^6^ TCID_50_		None (0/1)		
	1 × 10^8^ TCID_50_	i.p.	100% (1/1)	Weight loss, severe depression, anorexia, decreased activity	
	1 × 10^6^ TCID_50_		100% (1/1)		
NiV-M (isolate 199901924)	1 × 10^5^ TCID_50_	i.t./i.n.	100% (2/2) ^e^	Clinical signs not described	[119]
NiV-B (isolate 200401066)	1 × 10^5^ TCID_50_	i.t./i.n.	100% (2/2) ^e^	Clinical signs not described	

^a^ All animals treated with ribavirin pre- or post-infection. ^b^ Approximate average exposure dose across animals in each group. ^c^ Infused with IV saline as the control for mAb treatment. ^d^ Received adjuvant as the control for vaccine study. ^e^ Received nonspecific VSV vaccine.

**Table 7:** Experimental findings in other nonhuman primate models of henipavirus disease.

Species	Virus (Isolate)	Dose	Route	% Lethality	Clinical Disease	Reference
Cynomolgus Macaque	NiV-B (isolate 200401066)	5 × 10^5^ PFU	i.t./i.n.	None (0/3)	Pulmonary edema, abdominal breathing, fever (2/3), lymphadenitis (1/3)	[105]
	NiV-M (isolate 199902916)			None (0/3)	None	
	HeV (prototype)			None (0/2)	Agitation (1/2), increased aggressiveness (1/2), abdominal breathing (1/2)	
Common Marmoset	NiV-B (isolate 200401066)	6.33 × 10^4^ PFU	i.t./i.n.	100% (4/4)	Hyperventilation (4/4), anorexia (3/4), lethargy (2/4), open mouth breathing (1/4), hunched posture (1/4), hindlimb tremors (1/4)	[106]
Squirrel Monkey	NiV-M (UMMC1)	1 × 10^3^ PFU	i.v.	100% (2/2)	Uncoordinated motor movements, prostration, coma	[124]
		1 × 10^7^ PFU		50% (1/2)	Uncoordinated motor movements, prostration, coma, anorexia, depression	
		1 × 10^3^ PFU	i.n.	None (0/0)	Anorexia, seizure	
		1 × 10^7^ PFU		None (0/0)	Anorexia, seizure, ocular edema	

**Table 8:** Experimental findings in equine models of henipavirus disease.

Virus (Isolate)	Dose	Route	% Lethality	Clinical Disease	Reference
HeV (prototype)	N/A ^a^	i.v./i.n.	100% (2/2)	Fever, weakness, labored breathing, agitation	[126]
	2 × 10^7^ TCID_50_	i.v./i.n. aerosol	100% (2/2)	Mild temperature increase, labored breathing, depression	
HeV (Australia/Horse/2008/Redlands)	2 × 10^6^ TCID_50_	o.n.	100% (3/3)	Increased heart rate, depression, anorexia, serous nasal discharge, dyspnea, agitation, irritability, panting respiration	[127]

^a^ Animals challenged with a homogenate of blood, spleen, and lung from horses infected naturally in an HeV outbreak.

**Table 9:** Experimental findings in swine models of henipavirus disease.

Species (Age)	Virus (Isolate)	Dose	Route	% Lethality	Clinical Disease	Reference
Landrace	HeV (prototype)	6.6 × 10^7^ PFU	o.n.	100% (2/2)	Fever, inappetence, severe depression, respiratory distress, recumbency	[89]
Gottingen Minipigs	HeV (prototype)	2.0 × 10^7^ PFU	i.n.	N/A ^a^ (0/5)	Fever (1/5), mild depression, cough, respiratory distress, transient neurological signs	
Landrace (7–8-week-old)	NiV-M ^b^	2.5 × 10^5^ PFU	i.n.	N/A ^a^ (0/4)	Fever, lethargy, labored breathing (1/4), cough (1/4), unwilling to stand (1/4)	[131]
Landrace (9-week-old) ^c^	NiV-M ^b^	5 × 10^5^ PFU	i.n.	None (0/2)	Mild temperature increase	[132]
Landrace (9-week-old) ^c^	HeV (prototype)	5 × 10^5^ PFU	i.n.	None (0/1)	Mild temperature increase	
Landrace (4-week-old)	NiV-M (human isolate)	2.5 × 10^5^ PFU	o.n.	16% (1/6)	Transient temperature increase, exudative epidermitis (1/6), mild to severe depression (3/6), shivering, unwilling to stand (3/6), inappetence, shiver, hunched posture (2/4), cough (2/6), increased respiratory rate	[134]
Landrace (4–6-week-old)	NiV-M ^b^	2.5 × 10^5^ PFU	i.n.	31% (5/16)	Not described	[135]
Conventional (6-week-old)	NiV-M	5 × 10^3^ TCID_50_	s.c.	66% (2/3)	Loss of consciousness, lateral recumbency, ataxia, unwillingness to stand, muscle fasciculations, nasal discharge, shivering, persistent cough upon stimulation, mild increase in temperature	[130]
			oral	None (0/3)	No signs attributed to NiV infection	
Landrace (4-week-old)	NiV-M (human isolate)	2.5 × 10^5^ PFU	o.n. + ocular	18% (2/11) ^d^	Wide stance, difficulty standing, unsteady balance, restless, lethargy, unwilling to walk, sawhorse stance, depression, severe shivering, seizure	[136]
Landrace (5–6-week-old)	rNiV-B ^e^	2.5 × 10^5^ PFU	o.n.	None (0/10)	None	[137]

^a^ Animals were sacrificed at specific time points but did show clinical signs prior to euthanasia. ^b^ Human isolate passaged through experimentally infected pigs. ^c^ Approximate age at the time of challenge. ^d^ Animals euthanized due to the severity of the disease; however, animals were sacrificed at scheduled time points per study design. ^e^ Recombinant NiV-B recovered from reverse genetics system.

**Table 10:** Experimental findings in canine models of henipavirus disease.

Species (Age)	Virus (Isolate)	Dose	Route	% Lethality	Clinical Disease	Reference
Unknown	HeV (prototype)	5 × 10^3^ TCID_50_	s.c.	None (0/2)	None	[55]
Beagle (5–8-month-old)	HeV (Australia/Horse/2008/Redlands)	2 × 10^6^ TCID_50_	o.n.	None (0/6)	Mild inappetence, mild conjunctivitis, tonsillar hyperplasia, transient mild temperature increase	[139]

**Table 11:** Experimental findings in feline models of henipavirus disease.

Species (Age)	Virus (Isolate)	Dose	Route	% Lethality	Clinical Disease	Reference
Domestic shorthair cat (12–24-month-old)	NiV-M	5 × 10^3^ TCID_50_	s.c.	100% (2/2)	Fever, increased respiratory rate, inappetence, depression	[125]
		5 × 10^2^ TCID_50_		100% (4/4)		
Domestic shorthair cat	NiV-M	5 × 10^3^ TCID_50_	o.n.	50% (1/2)	Fever, depression, increased respiratory rates, vomiting, decreased grooming, dyspnea, open mouth breathing	[130]
Unknown	HeV (prototype)	5 × 10^3^ TCID_50_	s.c.	100% (2/2)	Inappetence, increased respiratory rate	[55]
Unknown	HeV (prototype)	5 × 10^4^ TCID_50_	o.r.	100% (2/2)	Open mouth breathing, dyspnea	[144]
Unknown (8-month-old)	HeV (prototype)	1 × 10^3.6^ TCID_50_	o.r.	100% (2/2)	Depression, fever, increased respiratory rate	[143]
			i.n.	100% (2/2)		
			s.c.	100% (2/2)		
		N/A	Contact ^a^	50% (1/2)		
Unknown	HeV (prototype)	5 × 10^3^ TCID_50_	s.c.	100% (2/2)	Not described	[86]

^a^ Naïve cat housed with an animal infected via the s.c. route.

## Data Availability

No new data were created or analyzed in this study. Data sharing is not applicable to this article.
